# Global, regional, and national burden of cardiovascular diseases attributable to secondhand smoke, 1990–2021

**DOI:** 10.3389/fpubh.2025.1642692

**Published:** 2026-01-14

**Authors:** Yunfeng Yu, Keke Tong, Xiangning Huang, Yuman Yin, Siyang Bai, Chenlu Guo, Liangjing Liu

**Affiliations:** 1 School of Medicine, Jishou University, Jishou, China; 2 Department of Endocrinology, The First Hospital of Hunan University of Chinese Medicine, Changsha, China; 3 School of Traditional Chinese Medicine, Hunan University of Chinese Medicine, Changsha, China

**Keywords:** secondhand smoke, cardiovascular diseases, global burden of disease, age-standardized rate, socio-demographic index

## Abstract

**Objective:**

This study assessed the global burden and trends of cardiovascular diseases attributable to secondhand smoke (CVD-SHS) from 1990 to 2021.

**Methods:**

The global burden of disease (GBD) database was utilized to analyze estimated annual percentage change (EAPC), age-standardized mortality rates (ASMR), age-standardized DALY rates (ASDR), disability-adjusted life years (DALYs), and deaths due to CVD-SHS. Subsequently, further analysis was conducted by region, age group, sex, and socio-demographic index (SDI). Finally, Spearman correlation analyses were used to assess the correlation of ASDR and ASMR with SDI.

**Results:**

From 1990 to 2021, global CVD-SHS deaths and DALYs increased by 34.5 and 23.1%, respectively, while ASMR and ASDR decreased by 41.8 and 42.0%. In 2021, CVD-SHS deaths totaled 694,692 (ASMR 8.31/100,000), with DALYs at 16,674,552 (ASDR 194.59/100,000). Regionally, the highest ASDR and ASMR were observed in low-middle and middle SDI regions, with minimal reductions in low SDI regions. Correlation analysis indicated that ASMR and ASDR, as well as their EAPCs, were negatively correlated with SDI. Moreover, CVD-SHS burden was higher in males and older age groups, predominantly affecting those aged 35 and above in lower SDI regions, and those aged 65 and above in high SDI regions.

**Conclusion:**

From 1990 to 2021, the global, regional, and national burden of CVD-SHS showed a paradoxical trend: while ASMR and ASDR declined, the absolute number of deaths and DALYs continued to rise. The burden and its growth rate were negatively correlated with SDI, with the highest impacts observed in low- and middle-SDI regions, males, and older populations. Moreover, stroke-SHS showed a stronger negative correlation with SDI than IHD-SHS, suggesting subtype-specific disparities. These findings highlight the persistent and uneven burden of CVD-SHS worldwide and underscore the need for targeted, region- and disease-specific prevention strategies.

## 1 Introduction

Cardiovascular diseases (CVD) are the leading cause of mortality and reduced life expectancy worldwide (1). These conditions include atrial fibrillation, rheumatic heart disease, cardiomyopathy, hypertensive heart disease, stroke, and ischemic heart disease (IHD) (2, 3). Globally, the number of deaths from CVD increased from 12.33 million in 1990 to 19.45 million in 2021, representing rose by 57.5% (4). In 2021, there were approximately 227.8 million IHD cases and 100.5 million stroke cases, making them the most prevalent forms of CVD (5). This trend is particularly alarming in China, where CVD deaths and incidence rates increased by 89.1 and 132.8%, respectively, from 1990 to 2019 (6). These findings underscore the global burden of CVD and highlight the importance of identifying and controlling its associated risk factors.

Established modifiable risk factors for CVD include active smoking, hypertension, dyslipidemia, diabetes mellitus, obesity, and physical inactivity (7). While these risk factors are well recognized, growing evidence suggests that exposure to secondhand smoke (SHS) also substantially contributes to CVD risk among non-smokers (8). According to the World Health Organization (WHO), there are approximately 1.3 billion smokers worldwide, leading to over 8 million deaths each year, including around 1.3 million deaths among non-smokers exposed to SHS (9). Unlike active smoking, SHS affects large numbers of non-smokers through involuntary exposure in homes, workplaces, and public spaces, resulting in considerable population-level harm even when individual relative risks are modest (9). Early epidemiological studies indicated that passive smoking increases the risk of CVD and mortality (10). Subsequent research has shown that SHS exposure raises the risk of IHD by 39%, stroke by 36%, hypertension by 28%, myocardial infarction by 50%, and non-specific CVD by 50% (11). Furthermore, SHS has been reported to cause a 22% increase in overall CVD risk among non-smokers, with the risk positively correlated with exposure dose (8). Conversely, implementation of smoke-free legislation has been associated with a 9–10% reduction in CVD risk and hospital admissions (12). Mechanistic studies suggest that SHS may contribute to CVD through impaired platelet and endothelial function, oxidative stress, vascular inflammation, and disruption of myocardial oxygen homeostasis (13). Collectively, these findings indicate that SHS is an important but often underestimated contributor to the global CVD burden.

Previous studies have estimated the burden of CVD attributable to SHS (CVD-SHS) using the global burden of disease (GBD) or similar frameworks up to 2019, revealing increasing absolute numbers of deaths and disability-adjusted life years (DALYs) (14–16). The release of the GBD 2021 dataset, however, allows for a more comprehensive and up-to-date assessment covering the period from 1990 to 2021 (17). This enables analysis of estimates for 204 countries and territories, as well as evaluation of the associations between CVD-SHS burden and the socio-demographic index (SDI). It also provides the opportunity to explore age- and sex-specific trends that were not fully characterized in earlier studies. Accordingly, using the GBD 2021 database, we assessed the global, regional, and national burden of CVD-SHS from 1990 to 2021. We analyzed deaths, DALYs, age-standardized rates, and estimated annual percentage changes (EAPCs), and further examined variations across SDI levels, regions, age groups, and sexes to inform targeted prevention and health policy strategies.

## 2 Methods

### 2.1 Data sources

The dataset for this study was derived from the GBD 2021 database^1^. The GBD 2021 database serves as a comprehensive and standardized repository of information on diseases, injuries, and risk factors across 204 countries and territories from 1990 to 2021. These countries and territories are grouped into 21 regions and seven super-regions, as defined by the GBD study. These regions and super-regions consist of geographically proximate countries and territories that exhibit epidemiological similarities and share comparable patterns in the distribution of causes of death (18).

SHS exposure in the GBD 2021 framework is defined as current exposure to tobacco smoke from other individuals in domestic environments, workplaces, or public spaces. In alignment with the standardized GBD methodology, SHS exposure was quantified using a multidimensional approach that incorporated self-reported exposure duration and frequency, biochemical markers of tobacco smoke metabolites, and environmental measurements of airborne tobacco smoke concentration (19). Participants were classified into exposure levels based on validated thresholds: non-exposed (no reported or measured smoke contact), low exposure (intermittent or occasional contact with environmental tobacco smoke), and high exposure (consistent and prolonged interaction with smoke in the surrounding environment). The operational definition accounted for both domestic and occupational exposure, considering variations in smoke density, exposure duration, and proximity to active smokers. Exposure assessment employed standardized and validated questionnaires across diverse demographic and geographic populations, ensuring measurement consistency and comparability across subgroups. The GBD study employs advanced statistical techniques, including geospatial Gaussian process regression models and Bayesian meta-regression, to generate internally consistent estimates. These models are adjusted for potential confounders and comorbidities to minimize bias from other risk factors such as air pollution, noise, and psychosocial stress (19).

In the context of SHS as an exposure, the CVD dataset included two subtypes: ischemic heart disease (IHD) and stroke, both classified according to the International Classification of Diseases, 10th Revision (ICD-10). The ICD-10 codes for IHD are I20–I25, representing conditions that affect the coronary arteries—typically due to atherosclerosis—resulting in angina, myocardial infarction, or ischemic cardiomyopathy. Stroke is coded as I64 and defined as a rapid onset of focal disturbance of cerebral function persisting for more than 24 h or leading to death. As the data used in this study were entirely derived from the publicly accessible GBD 2021 dataset, no additional ethical approval was required.

### 2.2 Statistical analysis

All analyses and visualizations were conducted using R software (version 4.4.1). We reported the global, regional, and national burdens of CVD-SHS using the number of deaths, DALYs, age-standardized mortality rate (ASMR), and age-standardized DALY rate (ASDR). Temporal trends were assessed using the EAPCs in ASMR and ASDR. Specifically, the number of deaths refers to those caused by disease or injury in the year. DALY represents the combined total of years of life lost due to premature death caused by disease and years of life lost due to disability, reflecting overall influence of disease or injury on the health of the population. ASMR refers to the age-standardized rate of deaths per 100,000 population, and ASDR refers to the age-standardized rate of DALYs per 100,000 population. We calculated the ASMR and ASDR using the following formula: $\mathrm{ASR}=\sum_{i=1}^{A}a_{i}w_{i}/\sum_{i=1}^{A}w_{i}\times 100,000$ , where *a_i_* represents the *i-*th age group and *w_i_* is the number (or proportion) of the population in the same age group in the GBD world standard population (20). All estimates are presented with 95% uncertainty intervals (UIs) derived from the GBD 2021 study framework. The EAPC, which represents the average annual percentage change in ASR, was calculated using the formula EAPC = 100 × (exp (*β*) – 1) and was presented along with its values and 95% confidence intervals (CI) (20). When EAPC > 0, ASMR or ASDR shows an upward trend; when EAPC < 0, ASMR or ASDR shows a downward trend.

Subsequently, we analyzed the ASMR and ASDR of CVD-SHS across different regions, development levels, sexes, and age groups. Specifically, the subgroup analysis of regions assessed the burden across 21 regions and 204 countries or territories. The subgroup analysis based on SDI categorized countries or territories into five SDI intervals and assessed the correlation between disease burden and SDI using Spearman’s rank correlation coefficient (*ρ*). Spearman’s correlation was selected instead of Pearson’s correlation because the data for SDI and age-standardized rates did not fully satisfy the assumptions of normality and linearity required for Pearson’s method. Spearman’s approach evaluates the strength and direction of a monotonic relationship between two ranked variables and is therefore more robust to non-linear associations and outliers. In this analysis, a *ρ* value close to 1 indicates a strong positive monotonic correlation, whereas a ρ value close to −1 indicates a strong negative monotonic correlation. A *p* value < 0.05 was considered statistically significant. The sex subgroup analysis assessed the disease burden separately for males and females. The age subgroup analysis divided the population into 15 age groups, each spanning 5 years, and reported the disease burden for individuals aged 25 and older. Furthermore, we simultaneously analyzed the burden and temporal trends of the two subtypes (ischemic heart disease attributable to secondhand smoke [IHD-SHS] and stroke attributable to secondhand smoke [stroke-SHS]), as well as their distribution in different SDI, sexes, and age subgroups.

## 3 Results

### 3.1 Global, regional, and national burden

#### 3.1.1 CVD-SHS

From 1990 to 2021, the global number of deaths from CVD-SHS increased from 516,603 (95% UI 380,624–670,267) to 694,692 (95% UI 493,036–903,631), while the ASMR decreased from 14.27 (95% UI 10.37–18.66) per 100,000 population to 8.31 (95% UI 5.92–10.82) per 100,000 population, with an EAPC of −1.91 (95% CI −2.01 to −1.82). The global DALYs of CVD-SHS increased from 13,544,948 (95% UI 9,898,715–17,369,518) to 16,674,552 (95% UI 11,986,655–21,409,778), while the ASDR decreased from 335.27 (246.04–432.01) per 100,000 population to 194.59 (139.78–250.16) per 100,000 population, with an EAPC of −1.94 (95% CI −2.04 to −1.84).

From 1990 to 2021, the region with the slowest decline in ASDR and ASMR was Oceania, with EAPCs of −0.44 (95% CI −0.48 to −0.39) and −0.44 (95% CI −0.49 to −0.39), respectively; the region with the fastest decline in ASDR and ASMR was Australasia, with EAPCs of −5.30 (−5.41 to −5.20) and −5.75 (−5.84 to −5.66), respectively. In 2021, the region with the highest ASMR was Central Asia at 19.52 (95% UI 13.78–26.79) per 100,000 population, and the region with the highest ASDR was Oceania at 415.08 (95% UI 268.76–574.25) per 100,000 population. In contrast, the region with the lowest ASDR and ASMR were Australasia at 31.68 (95% UI 20.03–46.28) per 100,000 population and 1.15 (95% UI 0.71–1.72) per 100,000 population, respectively. The burden and trends of CVD-SHS in the 21 regions are presented in Tables 1, 2.

**Table 1:** Death cases and ASMR of CVD-SHS and its temporal trends from 1990 to 2021.

Location	Death cases (95% UI)		ASMR (95% UI)		1990–2021 EAPCs (95% CI)
	1990	2021	1990	2021	
Global	516,603 (380,624–670,267)	694,692 (493,036–903,631)	14.27 (10.37–18.66)	8.31 (5.92–10.82)	−1.91 (−2.01 to −1.82)
SDI					
High SDI	93,835 (68,943–120,959)	52,429 (37,101–70,160)	8.68 (6.38–11.17)	2.48 (1.79–3.26)	−4.31 (−4.45 to −4.17)
High-middle SDI	172,123 (123,655–224,289)	207,513 (144,503–272,062)	19.86 (14.11–26.14)	10.78 (7.52–14.13)	−2.24 (−2.52 to −1.97)
Middle SDI	153,430 (111093–200,554)	267,826 (188160–352,447)	17.45 (12.4–23.12)	11.09 (7.67–14.77)	−1.55 (−1.61 to −1.48)
Low-middle SDI	78,380 (56,213–102,173)	137,296 (98,273–177,115)	14.02 (10.09–18.49)	10.21 (7.27–13.34)	−1.07 (−1.13 to −1.02)
Low SDI	18,035 (12,907–23,790)	29,023 (20,162–38,298)	8.76 (6.23–11.62)	6.26 (4.33–8.36)	−1.22 (−1.32 to −1.13)
Central Europe– eastern Europe– and central Asia					
Central Asia	11,118 (7,863–14,972)	13,490 (9,652–18,507)	26.06 (18.38–35.37)	19.52 (13.78–26.79)	−1.22 (−1.55 to −0.88)
Central Europe	34,986 (25,247–45,930)	19,769 (13,475–26,187)	26.07 (18.63–34.41)	8.66 (5.95–11.48)	−3.92 (−4.08 to −3.76)
Eastern Europe	59,741 (42,475–79,457)	44,870 (31,369–60,734)	24.15 (17–32.39)	12.82 (9.03–17.35)	−2.63 (−3.34 to −1.91)
High income region					
Australasia	1,511 (895–2,254)	617 (377–940)	6.69 (3.97–9.94)	1.15 (0.71–1.72)	−5.75 (−5.84 to −5.66)
High-income Asia Pacific	13,968 (9,687–18,574)	7,766 (5120–10,883)	7.72 (5.39–10.36)	1.5 (1.03–2.02)	−5.58 (−5.7 to −5.46)
High-income North America	25,918 (18,914–33,581)	13,928 (9,933–18,345)	7.53 (5.54–9.68)	2.21 (1.59–2.9)	−4.34 (−4.53 to −4.15)
Southern Latin America	5,197 (3,472–7,266)	2,821 (1,909–4,007)	12.04 (8.01–16.96)	3.22 (2.18–4.57)	−4.06 (−4.14 to −3.99)
Western Europe	43,677 (31,541–56,396)	15,482 (1,0380–20,907)	7.8 (5.63–10.08)	1.56 (1.09–2.09)	−5.37 (−5.49 to −5.25)
Latin America and Caribbean					
Andean Latin America	730 (487–964)	743 (481–1,045)	3.6 (2.37–4.77)	1.26 (0.82–1.77)	−3.96 (−4.33 to −3.59)
Caribbean	2,770 (1,815–3,739)	2,577 (1,696–3,607)	11.56 (7.58–15.7)	4.73 (3.11–6.61)	−3.25 (−3.42 to −3.08)
Central Latin America	4,875 (3,571–6,308)	6,818 (4,696–8,914)	6.48 (4.64–8.51)	2.79 (1.93–3.65)	−3.06 (−3.33 to −2.79)
Tropical Latin America	13,019 (9,193–17,047)	9,630 (6,697–12,972)	15.49 (10.88–20.38)	3.77 (2.62–5.08)	−4.7 (−4.84 to −4.56)
North Africa and Middle East					
North Africa and Middle East	41,772 (30,299–54,068)	67,355 (47,345–90,414)	28.51 (20.51–37.32)	16.73 (11.86–22.55)	−1.86 (−1.92 to −1.79)
South Asia					
South Asia	62,552 (45,489–81,854)	121,934 (87,384–159,342)	11.85 (8.54–15.58)	8.86 (6.31–11.68)	−1.12 (−1.19 to −1.04)
Southeast Asia– east Asia– and Oceania					
East Asia	145,222 (102,198–193,497)	278,060 (191,218–379,379)	21.39 (14.96–28.69)	14.53 (9.84–20.09)	−1.16 (−1.33 to −1)
Oceania	484 (307–665)	1,139 (735–1,582)	18.77 (12.01–25.39)	16.79 (10.93–23.11)	−0.44 (−0.49 to −0.39)
Southeast Asia	37,410 (26,696–49,016)	70,479 (48,289–93,883)	16.29 (11.63–21.74)	11.79 (8.12–15.65)	−1.09 (−1.18 to −1.01)
Sub–Saharan Africa					
Central Sub-Saharan Africa	1,173 (771–1,657)	2,020 (1,288–2,875)	5.88 (3.88–8.27)	4.15 (2.64–5.96)	−1.41 (−1.53 to −1.3)
Eastern Sub-Saharan Africa	3,958 (2,704–5,354)	5,134 (3,593–6,917)	8.68 (5.83–11.58)	3.13 (2.15–4.27)	−2.15 (−2.24 to −2.06)
Southern Sub-Saharan Africa	2,223 (1,515–2,939)	3,268 (2,287–4,309)	5.58 (3.8–7.47)	6.25 (4.32–8.28)	−0.97 (−1.35 to −0.6)
Western Sub-Saharan Africa	4,297 (2,994–5,666)	6,791 (4,575–9,044)	5.31 (3.67–7.12)	3.77 (2.54–5.07)	−1.28 (−1.46 to −1.1)
Cause					
Ischemic heart disease	287,517 (217,308–363,660)	414,640 (307,780–528,092)	8.01 (6.02–10.16)	4.97 (3.68–6.34)	−1.63 (−1.7 to −1.55)
Stroke	229,086 (159,150–307,214)	280,052 (185,101–375,639)	6.26 (4.38–8.48)	3.34 (2.21–4.48)	−2.31 (−2.44 to −2.18)

CVD-SHS, cardiovascular diseases attributable to secondhand smoke; ASMR, age-standardized mortality rate; EAPC, estimated annual percent changes.

**Table 2:** DALYs and ASDR of CVD-SHS and its temporal trends from 1990–2021.

Location	DALYs (95% UI)		ASDR (95% UI)		1990–2021 EAPCs (95% CI)
	1990	2021	1990	2021	
Global	13,544,948 (9,898,715–17,369,518)	16,674,552 (11,986,655–21,409,778)	335.27 (246.04–432.01)	194.59 (139.78–250.16)	−1.94 (−2.04 to −1.84)
SDI					
High SDI	2,252,307 (1,658,664–2,902,476)	1,206,763 (870,598–1,582,691)	213.02 (157.28–274.68)	67.52 (49.33–87.81)	−3.93 (−4.07 to −3.79)
High-middle SDI	4,152,430 (3,055,696–5,331,507)	4,396,617 (3,148,597–5,653,919)	428.25 (312.49–552.22)	229.53 (164.38–294.16)	−2.33 (−2.62 to −2.03)
Middle SDI	4,249,474 (3,086,699–5,527,109)	6,365,116 (4,578,060–8,263,320)	396.76 (287.54–517.96)	240 (171.62–313.15)	−1.73 (−1.78 to −1.68)
Low-middle SDI	2,326,804 (1,684,349–3,006,194)	3,827,736 (2,747,763–4,903,519)	349.04 (249.69–453.56)	251.37 (179.68–321.79)	−1.12 (−1.18 to −1.06)
Low SDI	544,709 (389,627–713,672)	864,351 (603,688–1,132,977)	220.4 (158.13–287.44)	152.84 (106–202.25)	−1.39 (−1.5 to −1.28)
Central Europe– eastern Europe– and central Asia					
Central Asia	270,840 (192,565–359,918)	320,302 (230,206–435,159)	573.64 (406.8–762.83)	399.28 (286.91–543.74)	−1.59 (−1.97 to −1.21)
Central Europe	806,663 (593,457–1,035,694)	381,641 (269,031–502,437)	563.8 (412.66–722.44)	183.14 (130.61–239.95)	−4.01 (−4.17 to −3.85)
Eastern Europe	1,312,584 (959,527–1,727,272)	955,464 (681,237–1,267,255)	492.87 (360.6–648.58)	286.46 (205.65–377.98)	−2.39 (−3.13 to −1.64)
High income region					
Australasia	36,654 (22,426–53,432)	14,589 (9,138–21,451)	162.14 (99.46–236.39)	31.68 (20.03–46.28)	−5.3 (−5.41 to −5.2)
High-income Asia Pacific	334,083 (235,814–433,989)	160,373 (110,112–216,687)	170.23 (119.68–222.63)	42.16 (29.08–56.34)	−4.78 (−4.9 to −4.67)
High-income North America	633,091 (467,174–808,544)	349,045 (253,982–458,332)	193.73 (142.93–247.55)	62.35 (45.43–81.89)	−3.98 (−4.15 to −3.82)
Southern Latin America	129,990 (86,934–180,273)	69,723 (47,931–98,822)	285.06 (190.52–396.51)	83.37 (57.32–117.96)	−3.86 (−3.92 to −3.81)
Western Europe	997,697 (729,251–1,285,400)	323,908 (230,111–427,320)	189.2 (139.57–243.73)	40.74 (29.35–53.14)	−5.12 (−5.23 to −5.01)
Latin America and Caribbean					
Andean Latin America	21,740 (14,633–28,846)	20,722 (13,352–29,020)	93.82 (63.46–124.14)	33.35 (21.73–46.63)	−3.88 (−4.22 to −3.53)
Caribbean	64,227 (43,514–84,802)	58,557 (38,679–80,729)	246.95 (167.3–327.41)	109.25 (72.26–150.81)	−2.92 (−3.1 to −2.75)
Central Latin America	129,733 (95,735–166,724)	170,060 (119,012–222,060)	146.14 (106.65–188.19)	66.56 (46.41–86.91)	−2.92 (−3.19 to −2.64)
Tropical Latin America	369,863 (261,606–482,605)	264,674 (184,748–354,784)	375.62 (264.18–489.93)	101.12 (70.7–135.52)	−4.45 (−4.59 to −4.32)
North Africa and Middle East					
North Africa and Middle East	1,164,284 (859,751–1,482,688)	1,800,423 (1,281,039–2,361,640)	651.1 (471.61–840.74)	372.8 (263.8–495.5)	−1.96 (−2.01 to −1.92)
South Asia					
South Asia	1,884,799 (1,367,073–2,472,246)	3,395,170 (2,437,593–4,430,078)	296.75 (215.12–388.21)	218.18 (157.74–283.5)	−1.18 (−1.26 to −1.11)
Southeast Asia– east Asia– and Oceania					
East Asia	3,907,462 (2,766,180–5,158,361)	5,872,430 (4,169,474–7,796,237)	457.96 (321.87–607.63)	285.93 (201.81–380.8)	−1.48 (−1.6 to −1.36)
Oceania	15,257 (9,588–21,298)	35,355 (22,881–49,737)	463.68 (295.02–634.49)	415.08 (268.76–574.25)	−0.44 (−0.48 to −0.39)
Southeast Asia	1,108,566 (804,129–1,450,094)	1,953,315 (1,353,352–2,598,655)	398.76 (282.88–517.28)	286.24 (198.56–381.16)	−1.1 (−1.16 to −1.03)
Sub–Saharan Africa					
Central Sub-Saharan Africa	36,358 (23,737–51,207)	63,417 (40,997–90,161)	147.46 (97.79–207.52)	101.04 (64.44–144.51)	−1.49 (−1.6 to −1.38)
Eastern Sub-Saharan Africa	126,683 (87,189–172,485)	166,628 (114,451–222,814)	148.75 (101.83–199.87)	82.66 (57.7–110.99)	−2.19 (−2.28 to −2.1)
Southern Sub-Saharan Africa	69,331 (48,500–90,791)	94,699 (67,306–125,469)	228.31 (159.04–300.06)	153.46 (107.65–201.94)	−1.15 (−1.52 to −0.79)
Western Sub-Saharan Africa	125,044 (86,072–166,449)	204,056 (139,223–273,397)	132.28 (91.96–174.21)	91.83 (62.23–122.92)	−1.38 (−1.57 to −1.19)
Cause					
Ischemic heart disease	7,391,461 (5,617,539–9,201,415)	9,704,289 (7,262,275–12,131,044)	183.43 (139.05–228.23)	113.44 (84.68–141.93)	−1.68 (−1.77 to −1.59)
Stroke	6,153,487 (4,278,034–8,256,920)	6,970,263 (4,734,823–9,224,428)	151.84 (105.34–203.71)	81.15 (55.07–107.4)	−2.28 (−2.4 to −2.16)

CVD-SHS, cardiovascular diseases attributable to secondhand smoke; DALYs, disability-adjusted life years; ASDR, age-standardized DALY rate; EAPC, estimated annual percent changes.

**Figure 1:**
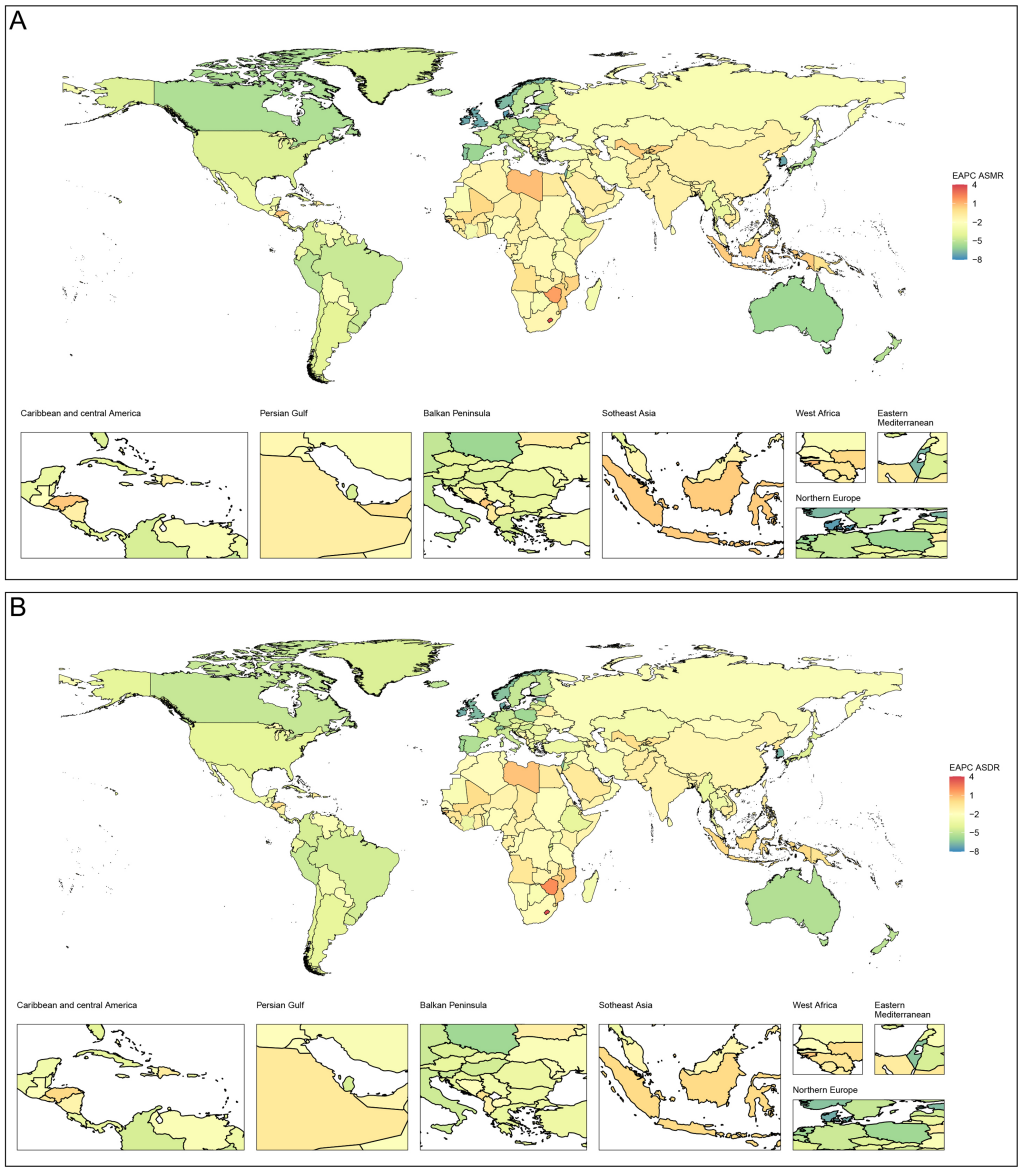
Global EAPCs of CVD-SHS in 204 countries or territories from 1990 to 2021. **(A)** EAPC of ASMR; **(B)** EAPC of ASDR. CVD-SHS, cardiovascular diseases attribute to secondhand smoke; EAPCs, estimated annual percentage changes; ASMR, age-standardized mortality rate; ASDR, age-standardized disability-adjusted life years rate.

**Figure 2:**
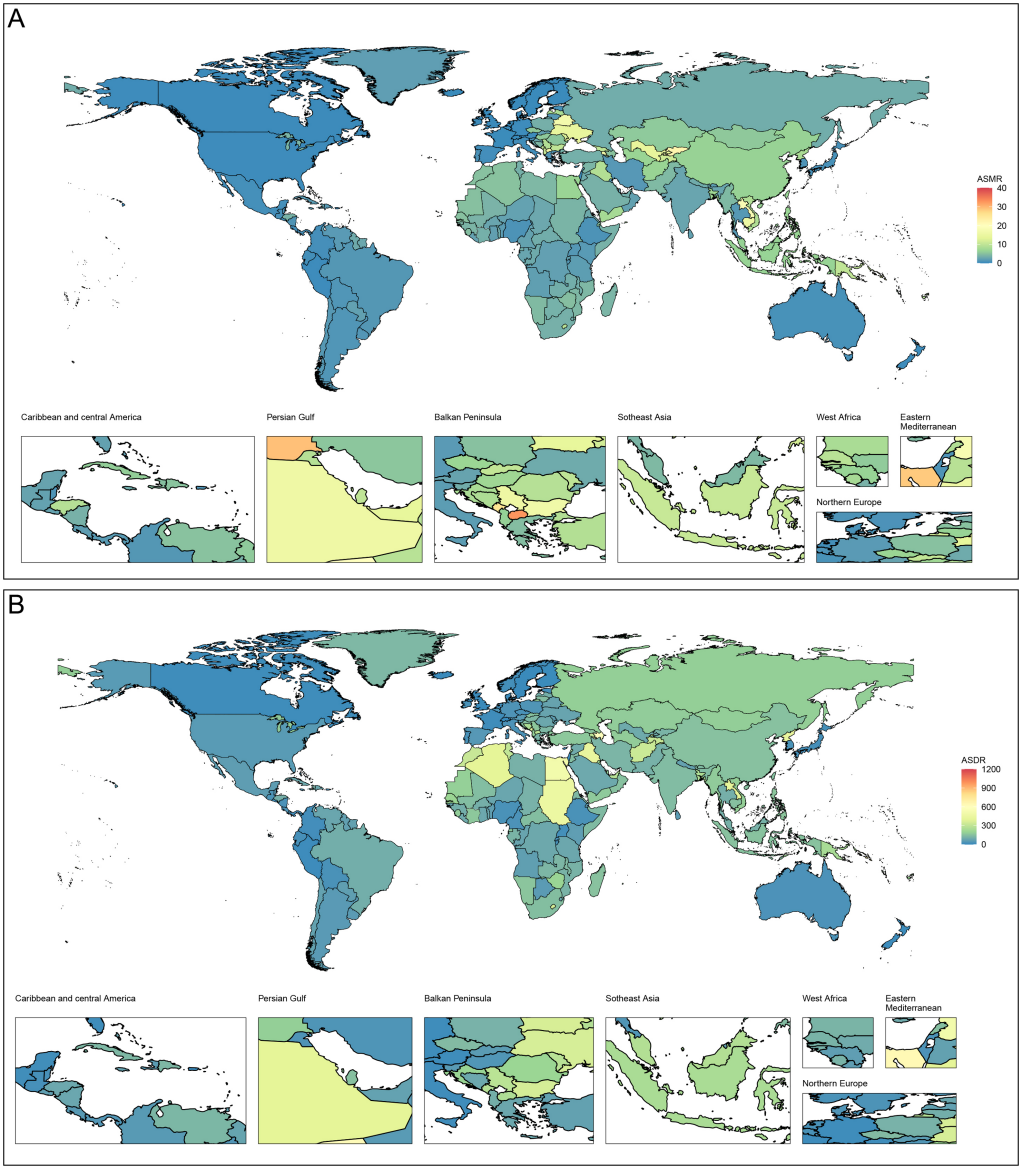
Age-standardized rates of CVD-SHS in 204 countries or territories in 2021. **(A)** ASMR; **(B)** ASDR. CVD-SHS, cardiovascular diseases attribute to secondhand smoke; ASMR, age-standardized mortality rate; ASDR, age-standardized disability-adjusted life years rate.

The top five countries with the highest ASMR were Nauru, North Macedonia, Turkmenistan, Azerbaijan, and Kiribati in 2021, with ASMRs of 37.26 (95% UI 23.81–51.07) per 100,000 population, 32.56 (95% UI 20.31–46.98) per 100,000 population, 30.39 (95% UI 20.30–41.53) per 100,000 population, 30.23 (95% UI 20.55–41.29) per 100,000 population, and 29.76 (95% UI 19.01–44.16) per 100,000 population, respectively. The top five countries with the highest ASDR were Nauru, Kiribati, Solomon Islands, Micronesia, and Turkmenistan, with 1026.4 (95% UI 654.73–1423.83) per 100,000 population, 788.10 (95% UI 503.10–1184.58) per 100,000 population, 700.82 (95% UI 441.65–1027.49) per 100,000 population, 677.23 (95% UI 429.21–964.30) per 100,000 population, and 672.06 (95% UI 446.07–916.40) per 100,000 population, respectively. The burden and trends of CVD-SHS in the 204 countries or territories are depicted in Figures 1, 2.

#### 3.1.2 CVD-SHS subtypes

From 1990 to 2021, both ASDR and ASMR of IHD-SHS and stroke-SHS exhibited a significant decline. The EAPCs for the former were −1.63 (95% CI −1.70 to −1.55) and −1.68 (95% CI −1.77 to −1.59), while for the latter, the EAPCs were −2.31 (95% CI −2.44 to −2.18) and −2.28 (95% CI −2.40 to −2.16), respectively. In 2021, the ASMRs of IHD-SHS and stroke-SHS were 4.97 (95% UI 3.68–6.34) and 3.34 (95% UI 2.21–4.48) per 100,000 population, respectively, while the ASDRs were 113.44 (95% UI 84.68–141.93) and 81.15 (95% UI 55.07–107.40) per 100,000 population. Regionally, the highest ASDR and ASMR for IHD-SHS were observed in Central Asia, at 286.24 (95% UI 200.37–395.80) and 14.58 (95%UI 10.02–20.22) per 100,000 population, respectively. Conversely, the ASMR and ASDR of stroke-SHS were highest in Oceania, at 8.98 (95% UI 5.61–12.58) and 220.05 (95% UI 136.94–310.22) per 100,000 population, respectively.

### 3.2 SDI disparity analysis

#### 3.2.1 CVD-SHS

**Figure 3:**
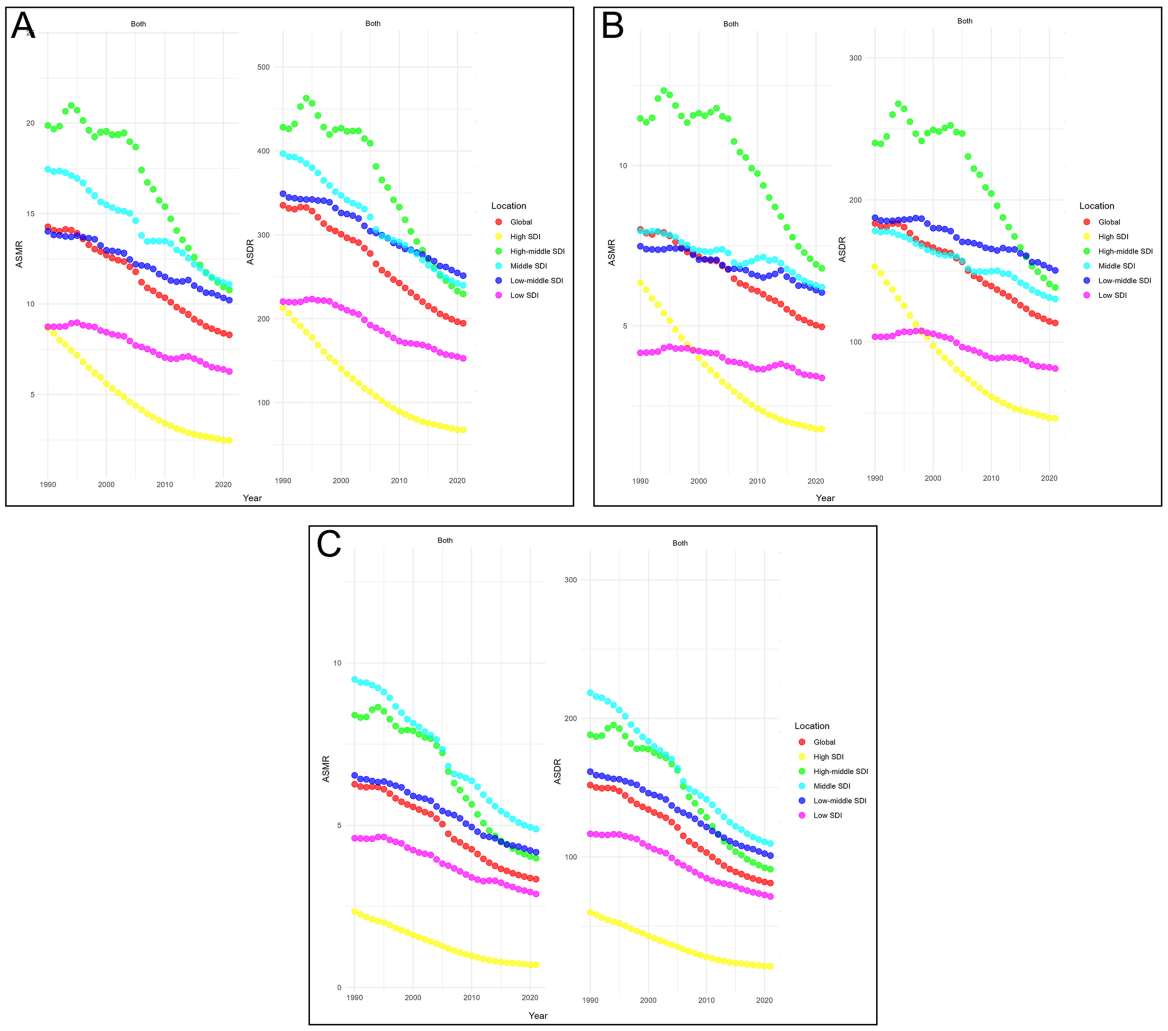
Temporal trends of age-standardized rates of CVD-SHS across different SDI quintiles. **(A)** CVD-SHS; **(B)** IHD-SHS; **(C)** stroke-SHS. SHS, secondhand smoke; CVD, cardiovascular diseases; IHD, ischemic heart disease; SDI, socio-demographic index; ASMR, age-standardized mortality rate; ASDR, age-standardized disability-adjusted life years rate.

From 1990 to 2021, both ASDR and ASMR decreased in every SDI region. Among them, the low SDI regions exhibited the slowest declines, with EAPCs of −1.22 (95% CI −1.32 to −1.13) and −1.39 (95% CI −1.50 to −1.28), respectively. In contrast, the high-middle SDI regions experienced the fastest declines, with EAPCs of −2.24 (95% CI −2.52 to −1.97) and −2.33 (95% CI −2.62 to −2.03), respectively. In 2021, the ASMR was highest in the middle SDI regions, at 11.09 (95% UI 7.67–14.77) per 100,000 population, while the ASDR was highest in the low-middle SDI regions, at 251.37 (95% UI 179.68–321.79) per 100,000 population, as illustrated in Figure 3A.

**Figure 4:**
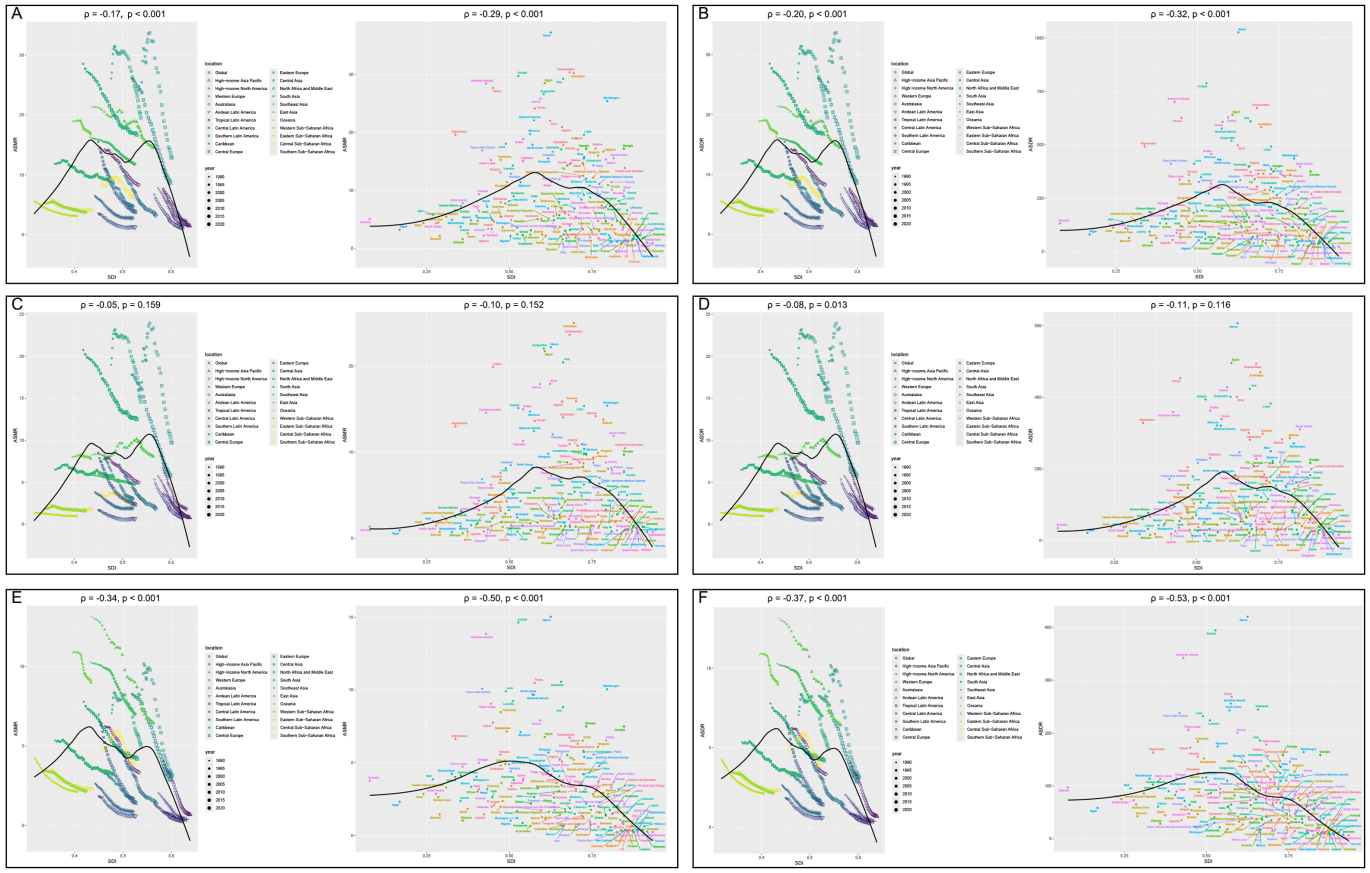
Correlation analyses between age-standardized rates of CVD-SHS and SDI from 1990 to 2021. **(A)** ASMR of CVD-SHS; **(B)** ASDR of CVD-SHS; **(C)** ASMR of IHD-SHS; **(D)** ASDR of IHD-SHS; **(E)** ASMR of stroke-SHS; **(F)** ASDR of stroke. SHS, secondhand smoke; CVD, cardiovascular diseases; IHD, ischemic heart disease; SDI, socio-demographic index; ASMR, age-standardized mortality rate; ASDR, age-standardized disability-adjusted life years rate.

**Figure 5:**
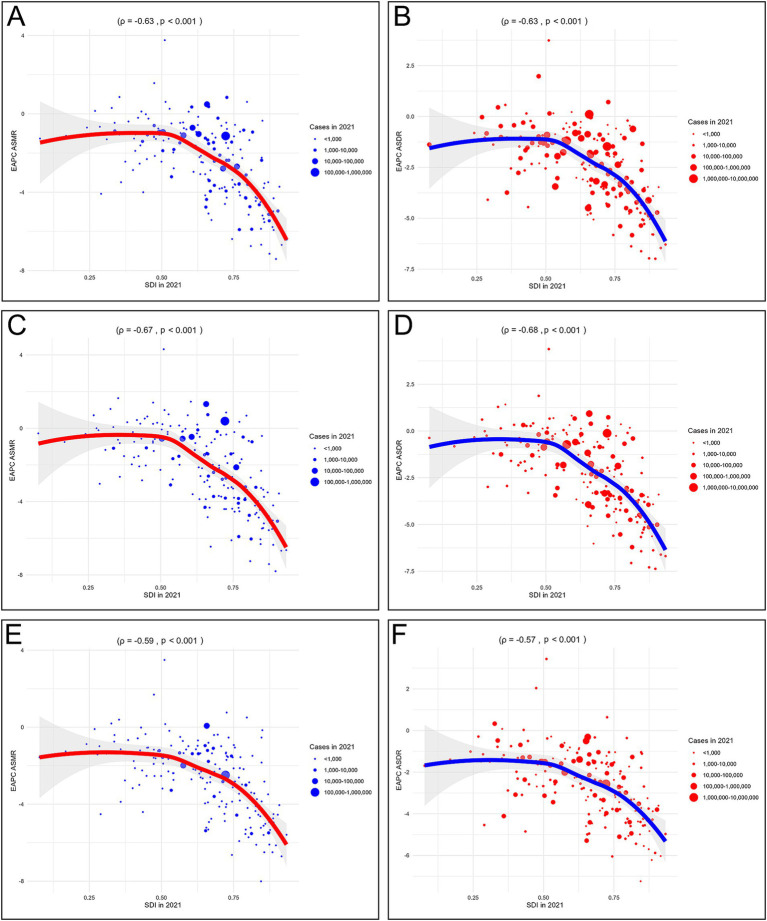
Correlation analyses between EAPCs of CVD-SHS and SDI in 2021. **(A)** EAPC of ASMR in CVD-SHS; **(B)** EAPC of ASDR in CVD-SHS; **(C)** EAPC of ASMR in IHD-SHS; **(D)** EAPC of ASDR in IHD-SHS; **(E)** EAPC of ASMR in stroke-SHS; **(F)** EAPC of ASDR in stroke-SHS. EAPCs, estimated annual percentage changes; SHS, secondhand smoke; CVD, cardiovascular diseases; IHD, ischemic heart disease; SDI, socio-demographic index; ASMR, age-standardized mortality rate; ASDR, age-standardized disability-adjusted life years rate.

Correlation analysis indicated that the ASMR in 22 regions (*ρ* = −0.17, *p* < 0.001) and 204 countries or territories (*ρ* = −0.29, *p* < 0.001) were negatively correlated with SDI, as depicted in Figure 4A. Similarly, the ASDR in 22 regions (*ρ* = −0.20, *p* < 0.001) and 204 countries or territories (*ρ* = −0.32, *p* < 0.001) were negatively correlated with SDI, as presented in Figure 4B. Moreover, the EAPCs of ASMR (*ρ* = −0.63, *p* < 0.001) and ASDR (*ρ* = −0.63, *p* < 0.001) were both negatively correlated with SDI in 2021, as shown in Figures 5A,B.

#### 3.2.2 CVD-SHS subtypes

From 1990 to 2021, the ASDR and ASMR of IHD-SHS and stroke-SHS decreased across all SDI region, with the slowest reduction observed in the low SDI regions. The ASMR of IHD-SHS was highest in the high-middle SDI regions in 2021, while the ASDR was highest in the low-middle SDI regions. Differently, the ASDR and ASMR of stroke-SHS were highest in the middle SDI regions, as presented in Figures 3B,C.

Correlation analysis revealed that the ASDR (*ρ* = −0.08, *p* = 0.013) of IHD-SHS in 22 regions was negatively correlated with SDI, while the ASMR of IHD-SHS in 22 regions was not related to SDI (*p* > 0.05). Similarly, both the ASMR and ASDR of IHD-SHS in 204 countries/territories were not related to SDI (*p* > 0.05), as shown in Figures 4C,D. Conversely, the ASMR (*ρ* = −0.34, *p* < 0.001; *ρ* = −0.37, *p* < 0.001) and ASDR (*ρ* = −0.50, *p* < 0.001; *ρ* = −0.53, *p* < 0.001) of stroke-SHS in 22 regions and 204 countries/territories were negatively correlated with SDI, as demonstrated in Figures 4E,F.

Furthermore, the EAPCs of ASMR (*ρ* = −0.67, *p* < 0.001) and ASDR (*ρ* = −0.68, *p* < 0.001) of IHD-SHS, as well as the EAPCs of ASMR (*ρ* = −0.59, *p* < 0.001) and ASDR (*ρ* = −0.57, *p* < 0.001) of stroke-SHS were negatively correlated with SDI in 2021, as presented in Figures 5C–F.

### 3.3 Sex and age disparity analysis

#### 3.3.1 CVD-SHS

**Figure 6:**
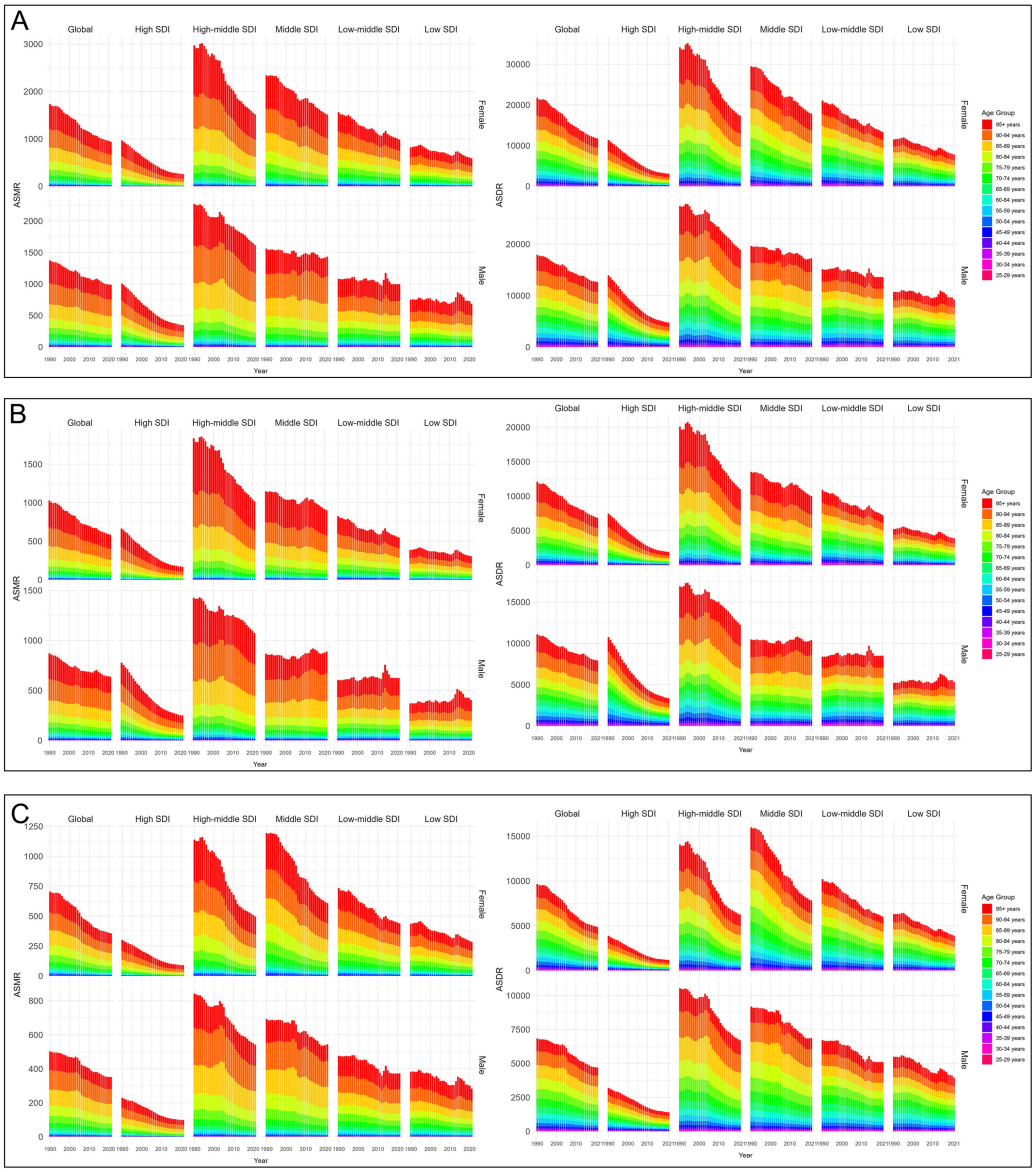
Age-standardized rates of CVD-SHS by sex and age groups from 1990 to 2021. **(A)** CVD-SHS; **(B)** IHD-SHS; **(C)** stroke-SHS. SHS, secondhand smoke; CVD, cardiovascular diseases; IHD, ischemic heart disease; SDI, socio-demographic index; ASMR, age-standardized mortality rate; ASDR, age-standardized disability-adjusted life years rate.

From 1990 to 2021, both the ASDR and ASMR for females and males showed downward trends, with a slower rate of decline observed in males compared to females. In 2021, the ASMR and ASDR for males were 8.87 (95% UI 6.29–11.54) and 209.52 (95% UI 149.11–270.23) per 100,000 population, respectively, which were higher than the ASMR at 7.83 (95% UI 5.54–10.39) per 100,000 population and ASDR at 180.47 (95% UI 130.67–233.55) per 100,000 population for females. Sex-related SDI analysis revealed that both for females and males, ASDR and ASMR were mainly derived from the high-middle, middle, and low-middle SDI regions, with the fastest reduction in ASMR and ASDR occurring in high-middle SDI regions, as illustrated in Figure 6A.

From 1990 to 2021, the ASDR and ASMR of all age groups decreased, with the slowest reduction observed in the 25–29 years age group. In 2021, the ASMR and ASDR of CVD-SHS were concentrated in the group over 65 years old, demonstrating a steady increase with advancing age. Age-related SDI analysis showed that the ASDR and ASMR in the low, low-middle, middle, and high-middle SDI regions were significantly distributed in the group over 35 years old. Conversely, in high SDI regions, ASDR and ASMR were predominantly concentrated in those aged 65 and above, as illustrated in Figure 6A.

#### 3.3.2 CVD-SHS subtypes

From 1990 to 2021, the ASDR and ASMR of IHD-SHS and stroke-SHS decreased for both males and females. In 2021, the ASDR and ASMR of IHD-SHS in males were 131.31 (95% UI 96.56–166.48) and 5.56 (95% UI 4.04–7.06) per 100,000 population, which were higher than the ASMR of 4.45 (95% UI 3.26–5.81) and ASDR of 96.42 (95% UI 73.08–122.25) per 100,000 population in females. In contrast, the ASDR and ASMR of stroke-SHS in males were 78.21 (95% UI 52.65–104.65) and 3.31 (95% UI 2.17–4.48) per 100,000 population, which were lower than the ASMR of 3.37 (95% UI 2.26–4.60) and ASDR of 84.05 (95% UI 56.59–111.41) per 100,000 population in females. Additionally, the ASMR and ASDR of IHD-SHS and stroke-SHS increased with age, and mainly came from the group aged 65 and above, as demonstrated in Figures 6B,C.

## 4 Discussion

### 4.1 Study significance and key findings

This study comprehensively assessed the global, regional, and national burden and temporal trends of CVD-SHS from 1990 to 2021, using the most recent GBD 2021 data. Whereas prior analyses evaluated SHS-related cardiovascular burden up to 2019 (14–16), our study extends the temporal coverage to 2021 to capture recent global trends. Moreover, this analysis incorporates systematic assessments across SDI levels, age groups, and sexes, offering a more detailed understanding of disparities that were incompletely characterized in earlier work. These enhancements strengthen the evidence base for targeted prevention and policy interventions addressing SHS exposure and cardiovascular health.

Our key findings are summarized below: First, from 1990 to 2021, DALYs and the number of deaths for CVD-SHS and its subtypes increased globally, while ASMR and ASDR decreased. Second, both ASDR and ASMR for CVD-SHS were negatively correlated with SDI. Specifically, the burden of stroke-SHS was negatively correlated with SDI, while the burden of IHD-SHS was not. Third, the EAPCs of ASDR and ASMR for CVD-SHS were negatively correlated with SDI, which was also found in both subtypes. Fourth, the ASDR and ASMR of CVD-SHS were more prominent among males and older age groups. The ASMR and ASDR in the low SDI, low-middle SDI, middle SDI, and high-middle SDI regions were significantly distributed in the group aged 35 and above, whereas in high SDI regions, ASDR and ASMR were predominantly concentrated in those aged 65 and above.

### 4.2 Global, regional, and national burden analysis

In the last 32 years, the overall global burden of CVD-SHS has shown an upward trend, with an increase in deaths and DALYs of 34.47 and 23.11%, respectively. However, the age-standardized burden of CVD-SHS per capita exhibited downward trends, with ASMR and ASDR decreasing by 41.77 and 41.96%, respectively. In the subtype analysis, IHD-SHS and stroke-SHS similarly indicated an upward trend in the overall burden and a downward trend in age-standardized burden per capita. This suggests that the increase in the overall burden of CVD-SHS may be caused by the rapid global population growth. Regionally, Oceania is the region with the slowest decline in ASDR and ASMR, while Australasia is the region with the fastest decline in ASDR and ASMR. Moreover, in 2021, Oceania is the region with the highest ASDR and the second highest ASMR, while Australasia is the region with the lowest ASDR and ASMR. This implies that Oceania remains a primary region afflicted by CVD-SHS, while Australasia has effectively and sustainably controlled the disease. In the GBD database, Oceania encompasses all countries in the region excluding Australasia, which is categorized within the high-income region.

We speculate that the observed differences in disease burden between these regions may be related to variations in tobacco consumption patterns, which are likely influenced by historical and cultural contexts. Specifically, it has been suggested that during the period of active colonization, colonists in Oceania introduced and promoted tobacco among indigenous populations through the glorification of its symbolism and its use as an alternative to cash payments, which may have contributed to the emergence of a commercial tobacco trade (21, 22). Within this historical and cultural framework, indigenous populations—already facing socioeconomic disadvantages—may have adopted and maintained certain tobacco use habits that contribute to the persistently high levels of tobacco consumption observed today (23). While these factors do not establish causality, they offer a possible sociocultural explanation for the regional disparities in CVD-SHS burden. In 2021, among the top five countries with the highest ASDR and ASMR rankings, Nauru, Kiribati, and the Solomon Islands were located in Oceania. Reports indicate that in 2020, smoking rates in these countries were 48.5, 40.6, and 36.5% (24), respectively, which were significantly higher than the rates in Australia (10.7%) and New Zealand (10.9%) (25, 26). This highlights the high smoking rate in Oceania, which directly determines the exposure risk of SHS. The Global Youth Tobacco Survey reveals that in the Oceania nation of Kiribati, the probabilities of adolescents being exposed to SHS at home and in public places were 66.9 and 69.8%, respectively (27). In another Oceania nation, the Solomon Islands, the probabilities of SHS exposure were 61.3% at home and 68.3% in public spaces (28). These findings explain why Oceania has become a major disaster region for CVD-SHS.

In Australasia, indigenous populations such as Australasia’s former Aboriginal and Torres Strait Islander peoples have much higher smoking and associated mortality rates than non-indigenous populations (29). However, following the implementation of various tobacco control measures, smoking prevalence and SHS exposure rates in Australasia have declined rapidly. In 2010, Australia added a series of tobacco tax programs that increased tobacco taxes by 25% (30). In 2012, Australia enacted and implemented the world’s first tobacco plain packaging laws, mandating uniform packaging with health warning images (31). Subsequently, 20 countries, including New Zealand, the United Kingdom, Norway, France, Canada, and Singapore, followed suit in passing similar legislation (32). In addition, Australia has specifically established the Tackling Indigenous Smoking Program to reduce tobacco consumption among the indigenous population (33). According to statistics, the daily smoking rate of the indigenous population decreased by 9.8% from 2018 to 2019 compared to 2004 to 2005 (34). This suggests that increasing tobacco taxes, restructuring tobacco packaging, and developing group-specific prevention and management policies have been effective in reducing tobacco consumption in Australasia, thereby fundamentally reducing the risk of SHS exposure. Therefore, we recommend that regions such as Oceania follow the example of Australia’s prevention and management policies to reduce tobacco consumption and the burden of CVD-SHS through tax, legal, and targeted programs.

Regarding subtypes, the highest ASDR and ASMR of stroke-SHS were in Oceania, while the highest ASDR and ASMR of IHD-SHS were in Central Asia. Notably, a significant portion of the IHD-SHS burden in Central Asia is attributed to inadequate tobacco control measures. For instance, Uzbekistan has yet to implement a ban on smoking in public areas, while Kyrgyzstan has experienced only a 1.4% decline in tobacco use from 2014 to 2019 following the introduction of tobacco control policies (35). These regions could benefit from the Australian government’s experience in managing and controlling tobacco consumption to effectively reduce the burden of IHD-SHS and stroke-SHS.

### 4.3 Analysis of differences based on SDI

Our findings indicate that both the ASDR and ASMR of CVD-SHS and their respective EAPCs are negatively correlated with SDI. Specifically, ASDR and ASMR in the low-middle SDI and middle SDI regions were persistently high and exhibited slow declines, whereas the burden in the high SDI region was small and decreased rapidly. This reflects that SDI determines factors, such as socioeconomic level and education, significantly impact the burden of CVD-SHS.

First, socioeconomic level influences CVD-SHS burden through income level and policies related to prevention and management. According to the WHO report on the global tobacco epidemic 2023, 80% of smokers reside in low- and middle-income countries (35). Specifically, in 2019, the top 10 countries with the highest number of smokers globally—China, India, Indonesia, the United States, Russia, Bangladesh, Japan, Turkey, Vietnam, and the Philippines—were mostly from the middle and low-middle SDI regions (36). In fact, the prevalence of tobacco products use is higher among populations with lower socioeconomic status (23, 37), which is more common in middle-income and lower-middle-income countries (38). It is reported that SHS exposure among non-smokers in middle-income and lower-middle-income countries is 3.7 times and 2.2 times higher than in high-income countries, respectively (39). Although the global prevalence of tobacco use declined from 32.7% in 2000 to 22.3% in 2020 following the adoption of the Framework Convention on Tobacco Control (FCTC) by WHO in 2003, the prevalence of tobacco use in low- and middle-income countries is still not optimistic (40, 41). Limited socio-economic levels make it difficult for low- and middle-income countries to provide strong support for cessation services, tobacco dependence treatment, and protection of the health and livelihoods of tobacco workers, as required by the FCTC (42). Moreover, lack of implementation experience, insufficient grassroots enforcement, and impracticality of existing policies have also affected tobacco control in these countries and regions (43). Together, these factors contribute to the high and slow decline in the burden of CVD-SHS in low SDI, low-middle SDI, and middle SDI regions. Conversely, smoking rates in high-income countries such as the United States, Iceland, Norway, Sweden, and Canada have all been declining at an annualized rate of 2% since 1980 (44), thereby reducing the disease burden associated with smoking and SHS at the root. Moreover, the average educational attainment in low SDI regions is lower, which similarly influences the burden and temporal trends of CVD-SHS. For instance, smoking rates are higher among individuals with lower educational attainment compared to those with higher education (45). Additionally, individuals with higher education are more receptive to tobacco control policies and exhibit significantly higher smoking cessation rates than their lower-educated counterparts (46). Consequently, these factors contribute to a lower rate of SHS exposure among the high-education group. Regarding this situation, a number of randomized controlled trial programs are planning SHS prevention and control education for children and pregnant women, and to assess the value of SHS prevention and control education for specific groups (47, 48).

Our findings revealed that although both IHD-SHS and stroke-SHS showed overall declines in ASMR and ASDR from 1990 to 2021, the patterns and magnitudes of these changes differed substantially. The burden of stroke-SHS exhibited a stronger negative correlation with SDI compared with IHD-SHS, suggesting that stroke is more sensitive to socioeconomic development and improvements in healthcare access. In contrast, the relatively weaker association between IHD-SHS and SDI may indicate persistent exposure to SHS-related metabolic and vascular risks even in higher-SDI regions. These subtype-specific differences imply that strategies to mitigate SHS-related cardiovascular burden should be disease-targeted: stroke prevention efforts may benefit most from strengthening public health infrastructure and smoking control in low-SDI areas, whereas IHD prevention may require broader cardiovascular risk management and continuous reduction of SHS exposure even in developed settings.

### 4.4 Analysis based on sex and age differences

Sex analysis showed that ASDR and ASMR of CVD-SHS were consistently higher in males than in females from 1990 to 2021, which was related to smoking prevalence and workplace exposure in males. Globally, the number of male smokers is approximately 4.7 times greater than that of females (49), making them the primary victims of SHS exposure. The high prevalence of smoking among males may be related to traditional conceptual perceptions, whereby male smoking is often perceived as masculinity and male traits. The tobacco industry has capitalized on this, promoting smoking as a symbol of male identity and adventurous spirit (50). Additionally, interpersonal factors, peer pressure, and perceived social pressure also have an impact on males’ smoking choices (51, 52). Furthermore, males’ workplace and social environments also increase their risk of SHS exposure (53). Therefore, some researchers have advocated for sex-specific strategies in addressing SHS exposure (54). First, it is essential to separate the sexed symbolism of tobacco to counteract the tobacco industry’s promotional advertisements. Second, smoking cessation interventions should be tailored to meet the distinct needs of males and females, focusing on reducing male cigarette socialization, relieving social pressure, and focusing on vulnerable females’ SHS exposure (55).

Age analyses revealed that the ASDR and ASMR in low, low-middle, middle, and high-middle SDI regions were significantly distributed in the group aged 35 and above, whereas ASMR and ASDR in the high SDI regions were significantly distributed only in the 65 years and older age group. First, the differences in disease burden among different SDI regions may be related to population structure. According to the WHO’s global report on tobacco use trends from 2000 to 2025, individuals aged 35 to 64 is the main tobacco user population globally (41), which provides an explanation for the significant distribution of ASMR and ASDR in the 35 to 64 year old age group in the above SDI regions. In contrast, the ASDR and ASMR in the high SDI regions have a significant distribution only in the age group of 65 years and above, which may be influenced by the aging of the population (56). Second, the lack of tobacco control and low prices equally affect the burden of disease in the 35 to 64 age group in the above-mentioned regions. In South-East Asia, transnational tobacco companies are taking over the pillars of economic growth and are using litigation and deception to counteract governments, owing to insufficient government experience and legal support (57). In countries such as Zimbabwe, Zambia, the United Republic of Tanzania, Malawi, and Mozambique—among the top five tobacco-producing nations in Africa—government efforts to formulate and implement tobacco control policies are frequently obstructed by the tobacco industry, which complicates the balance between tobacco control and economic objectives (58). Furthermore, tobacco taxes in most low- and middle-income African regions are significantly lower than in high-income regions (58), facilitating easier access to inexpensive tobacco products. These factors lead to significantly higher SHS exposure in the 35–64 year old group in low, low-middle, middle, and high-middle SDI regions than in the high SDI region, and ultimately affect the CVD-SHS burden. Therefore, we suggest that low- and middle-income countries should be alert to the economic aggression of the tobacco industry and learn from the tobacco control policies of high-income countries according to their own situation, such as appropriately raising tobacco taxes to reduce tobacco consumption of the population and increasing the financial expenditures for smoking cessation programs.

## 5 Limitations and prospects

This study is subject to several limitations: First, when SHS is considered as an exposure, the GBD database only provides burden data for two subtypes, IHD and stroke, and the burden of other CVD associated with SHS is unclear. Second, there are obvious gaps in the diagnostic level and data accuracy in different countries or territories, which may lead to biased evaluation results. Furthermore, the influencing factors of CVD-SHS summarized in this study are only speculations based on epidemiological data, and a clear causal relationship cannot be confirmed. In light of these limitations, we encourage future studies to adopt more rigorous data collection methods and standardized disease classification criteria to more accurately assess the burden and temporal trends of CVD-SHS in various regions. This approach will provide a solid foundation for developing effective prevention and management strategies.

## 6 Conclusion

Our study provides a comprehensive overview of the global, regional, and national burden of CVD-SHS from 1990 to 2021. Although the ASMR and ASDR of CVD-SHS have declined worldwide over the past three decades, the absolute number of deaths and DALYs has continued to increase, indicating a growing global burden. The disease burden and its growth rate were negatively correlated with the SDI, with the heaviest impacts observed in low- and middle-SDI regions, among males, and in older age groups. Importantly, the effects of SHS exposure differed by cardiovascular disease subtype. Stroke-SHS showed a stronger negative correlation with SDI than IHD-SHS, suggesting that improvements in socioeconomic development and healthcare access may have a greater impact on reducing SHS-related stroke burden than IHD burden. These findings highlight the persistent and uneven global burden of CVD-SHS and emphasize the urgency of implementing evidence-based, region-specific, and disease-specific strategies.

## Data Availability

The original contributions presented in the study are included in the article/supplementary material, further inquiries can be directed to the corresponding author.

## Footnotes

1
vizhub.healthdata.org
